# "The Hospital" Nursing Mirror

**Published:** 1898-01-01

**Authors:** 


"The Hospital" Nursing Mirror.

Being the Nursing Section of "The Hospital."

[Contributions for this Section of "The Hospital" should be addressed to the Editor, The Hospital, 28 & 29, Southampton Street, Strand, London, W.C., and should have the word "Nursing" plainly written in left-hand top corner of the envelope.]

### News from the Nursing World.

### A NEW YEAR'S GREETING.

We wish our readers a very Happy New Year. The cause of nursing becomes more and more organised as time goes by. There are now many admirable societies and guilds banding the members in an alliance greatly to the benefit of all. It is a nurse's own fault if she stands alone, and we would point out how very undesirable it is for her and for all that any nurse should do so. In the first place she is cut off from the mutual help such associations give, and, secondly, she deprives the association of the help she would be to it. There are now co-operations which ensure the nurse her earnings; there is also the Pension Fund to provide for her old age, the Sick Fund to tide her over sickness, and the Junius Morgan Benevolent Fund to help her when she cannot help herself. Then there is the Queen Victoria's Jubilee Nurses' Institute for district nurses, besides many other associations and institutions to meet the needs of nearly every kind of nurse. Nor must we forget the Nurses' (Commemoration) Club, which is a new departure for this year, and which will certainly offer social and educational advantages of a very valuable kind. Whilst wishing our readers a very prosperous year, we hope that they will not overlook the fact that much of their prosperity lies in loyal co-operation one with another.

### A CHAIRMAN AND HIS NURSES.

The Hon. Sydney Holland, chairman of the London Hospital, has given two lectures to the nurses of that institution during the month of December. The lectures were very characteristic in form, and contained much excellent advice and common sense. They have been printed by Whitehead, Morris, and Co. (Limited), 9, Fenchurch Street, E.C., and we are confident that they would prove of assistance and interest to the chairman of every hospital, who has the enterprise to obtain a copy, and to peruse it carefully. Certainly, the London hospital at the present crisis in its history is fortunate in possessing so energetic and enthusiastic a chairman as the author of these lectures.

### THERE IS NOTHING TO PAY.

Sometimes, when we have been fortunate enough to help our querist in some difficulty, we are asked if there is "anything to pay?" so we take the opportunity of this being the first number in the new year to tell our readers that there is no charge whatever for inserting news of appointments, presentations, or items of general interest, but that these announcements must of necessity be very brief, because there is not any room to spare. We are also pleased to answer queries put through the "Notes and Queries" column free of charge, but when the answer is required by post we are obliged to charge a fee of 2s. 6d. A moment's thought will show that this is necessary to guard ourselves against a host of irrelevant questions which would more than occupy one person's time to answer.

### NEW AND CONGENIAL WORK FOR WOMEN.

Since the barrack school and workhouse are confessed failures as far as the rearing of children, at any rate, is concerned, new methods are under discussion by parochial authorities, and the results of experiments and the trend of the opinion of those who know best about such things is towards boarding-out such pauper children as are suitable, and establishing the residue in cottage homes, presided over by a "mother," the "mothers," when possible, to be trained women of refinement, with a nurses' certificate. The cottage homes will probably be placed in neighbourhoods where there is a demand for young workers, and the number of inmates will average a dozen, principally girls. Boys, it seems, thrive well enough in larger establishments. This makes it almost certain that within a few years there will be a steady demand for "mother" nurses, and thus an opening at once congenial and useful will be given to women with the requisite qualifications. It is not improbable that many women in whom the vocation towards "mothering" is much stronger than towards nursing will be attracted to this work. And it is not too soon for such as have a bent that way to put themselves in training for it. It is a new calling, and will also give an opportunity to nurses of more mature age and experience, as obviously it cannot be desirable for very young women to live alone and rule so large a family.

### A PRACTICAL EXAMPLE.

Nurse Franklin, who has recently resigned her post in Romford Infirmary, has stated her reasons for doing so. It appears that her certificate was only for two and a half years, whilst a three-year certificate is necessary for advancement under the Local Government Board order. Miss Franklin therefore wrote to the authorities under which she trained stating her difficulty, and they immediately offered her work as "sister," and to grant her a three-year certificate after six months. She was, of course, glad to accept this practical solution of her difficulty, and in so doing has set an example that nurses in the same dilemma cannot do better than follow.

### A CHRISTMAS CATASTROPHE.

A very sad and painful incident occurred to one of the nursing staff of the Mill Road Infirmary, Liverpool, on Thursday 23rd ult. An entertainment had been arranged by the nurses, which took the form of waxworks, the performers being dressed in costumes. The unfortunate victim of the sad accident was Nurse Ashcroft, who had been a probationer in the hospital for eighteen months. Instead of entering the stage by the proper stage-door, Nurse Ashcroft had, in a moment of forgetfulness, climbed on to the stage from the recreation hall and stepped over the footlights. In doing so the art muslin drapery in which she was enveloped caught fire, and in a moment she was all in a blaze. Her fellow-nurses on the stage wrapped her up in a carpet, and in a short time extinguished the flames, but not before the poor nurse had been terribly burnt about the arms, face, and body. The medical superintendent, Dr. Nathan Raw, was in immediate attendance, and did everything possible for the girl's sufferings, but she sank and died from shock on Saturday night. The unfortunate affair has cast a gloom over the whole of the hospital, and all the festivities have been postponed. An inquest was held on Tuesday, and a verdict of accidental death returned.

### CAMBRIDGESHIRE'S THANK-OFFERING.

CAMBRIDGESHIRE's notable men resolved to celebrate Her Majesty's glorious reign by collecting and spending £5,000. They, however, reckoned without their subscribers, and a little over £1,800 remained after £500 had gone in rejoicings. Very sensibly they saw that, although £1,800 is not so goodly a sum as £5,000, yet excellent, if not showy, work can be done with it, and this is how they have decided to lay it out. They propose to purchase the house that the district nurses now occupy, and the one next it, to spend £100 in adapting the two houses to the needs of the nurses, and another £265 in furnishing them suitably, the balance—about £500—to be carried to the investment fund. Doubtless the nurses will be much pleased at the prospect of occupying a comfortable and suitable abode.

### WHY NOT A GREAT ROLL OF TRAINED NURSES?

IT will be seen on reference to page 236 of THE HOSPITAL for this week that th Prince of Wales's Hospital Fund Stamps and Stamp Albums are being utilised for a new Christmas game for children. It consists in offering every child a free photograph and the entrance of its name upon the Great Roll of Ministering Children, 100,000 in number, on production of one of the albums containing both the Hospital Stamps. We have been asked, Why not have a Great Roll for Trained Nurses? and we hand on the question to the nursing profession to answer. It would be highly interesting to have a complete album containing a photograph and autograph of every trained nurse in the Diamond Jubilee year. Still, nurses have many claims upon them, and we feel we must not urge the matter.

### THE NURSES' CO-OPERATION.

H.R.H. PRINCESS LOUISE (Marchioness of Lorne) has graciously consented to become patroness of the Nurses' Co-operation. The staff may well congratulate themselves on the honour done them by a Princess whose interest in nursing and nurses is well known. The Co-operation, which was founded in 1891, and incorporated in 1894, has a staff of 450 trained certificated nurses, and has offices at 8, New Cavendish Street, Portland Place.

### A BUILDING DEBT CANCELLED.

THE committee of the Kingston (Surrey) Nursing Association are to be congratulated upon the cancelling of the building debt on the new Nurses' Home in Birkenhead Avenue. A Jubilee gift of £100 from an anonymous lady, £50 from Mr. George Bolton, and £50 from Mr. Robert Norton complete the required sum.

### DERBYSHIRE ROYAL INFIRMARY.

THE Weekly Board of the Derbyshire Royal Infirmary, on the recommendation of the Nursing Committee, have decided to increase the training for future probationers from two years to three before granting a certificate of efficiency. Under the new scheme probationers will be taken without fee; they will receive no salary for the first year, £10 for the second, and £16 for the third. Indoor uniform will be provided for those in the second and third years. The staff of each ward will consist of a sister and three probationers, the old rank of staff nurse being abolished, the duties being performed by the third-year nurse, and the sisters will, as far as possible, be promoted from the senior probationers. Lectures will be given by members of the honorary medical staff, the matron, assistant matron, and housekeeper.

### GLASGOW SICK POOR AND PRIVATE NURSING ASSOCIATION.

THIS is a very large and seemingly popular association. There are 24 district and 37 private nurses at work on its staff. Nevertheless, the deficiency of £588 is a serious one, and it is quite time the committee bestirred themselves and put the finances of their society in order. Last year the deficit was greater, and, if this state of things continues, no amount of good work will make up for lack of business foresight in money matters. The resolution not to go into debt and to keep a sharp look out on the way the money goes is one of the best means to attract subscribers.

### SHORT ITEMS.

THE Selkirk district nurse (a Queen's nurse) has just finished a year of hard and successful work. Many of her patients have been nursed at the request of their medical attendant, and some at their own request. The nurse is well supported, and the Committee of Management appears to be both energetic and economical.—The Guardians of Grimsby have voted £5 to the Grimsby and District Nursing Association, which has shown that 51 persons in receipt of outdoor relief have been nursed by it during the year. Five pounds subscription is also promised to the Nursing Society, known as the Sisters of Peace, if the statistics are satisfactory.—The Guardians of Stourbridge are not eager to fall in with the requisition of the Local Government Board to appoint two extra nurses, in spite of the fact that an old lady of 60 has charge of 80 patients.—Dorchester District Nursing Association is one year old, and its financial troubles have already begun. Subscriptions have not come in so freely as they ought, and but for the kindness of the president, Mrs. Fellows, who has paid for the nurse's lodging, the association must have begun the new year in debt.—The Northampton Jubilee Nurses have received a good report from the president of Queen Victoria's Jubilee Institute.—Jarrow's Nursing Association seems popular. A variety entertainment, consisting of a promenade concert, *tableaux vivants,* and a dance took place in the Mechanics' Institute and was well attended.—The Hackney Vestry decided at their last meeting to appoint a lady sanitary inspector, whose special duty would be to look after the sanitary condition of the workshops, workrooms, and laundries where women and girls were employed.—Annan Jubilee Nursing Association pursues a quiet course of usefulness. The nurse does her work well. It is suggested that she be provided with a bicycle. A slightly larger number of cases were nursed, and the bank balance is pretty much the same.

### Lectures to Surgical Nurses.

By H. A. LATIMER, M.D. (Dunelm), M.R.C.S. of Eng., L.S.A. of London, Consulting Surgeon, Swansea Hospital; President of the Swansea Medical Society; Lecturer and Examiner of the St. John Ambulance Association, &c.

### XXI.—GRAVITY OF FRACTURES OF SPINAL AND PELVIC BONES AND OF THE FEMUR: COMPLICATIONS OF SUCH FRACTURES—THE PADDING OF SPLINTS AND THE RESULTS OF THEIR NEAT APPLICATION—BADLY SET, UNUNITED AND COMPLICATED FRACTURES.

THE gravity of fractures of the spinal bones, like that of fractures of the skull, lies in the fact that these bones enclose nerve structures, and that if injured or displaced they press upon and more or less completely interrupt the current of nerve force which that mass of nerve matter carries. When pressed upon, paralysis of the parts of the body below the injured portion of the spinal cord results, and we meet with loss of power and of feeling in any region which is controlled by parts of the cord situated beyond the seat of injury. It is in these cases of spinal injury that you, as nurses, have the greatest difficulty in warding off complications which are prone to arise; for the muscles which guard the bladder and bowel lose their power, and after the receipt of any serious injury the contents of these organs escape, and the sufferers are continually wetted and soiled by these discharges. Add to this the fact that by the withdrawal of nerve influence the skin in the paralysed area has become liable to die, and you will see with what rapidity bed-sores form, and how difficult it is to ward against them. The patients are nursed on water beds, and the utmost vigilance has to be exercised to keep them dry and to prevent these complications arising. As I shall tell you of bed sores, describe their mode of origin, and the means which you will adopt for preventing their formation, and for curing them when they have occurred, in my next lecture, I will say no more on this point now. But I must speak of the dribbling of urine which occurs in these paralytic cases, and explain how it arises. Water is expelled from the bladder by voluntary effort resulting from a feeling that the bladder is full, and should be emptied; muscles are spread over its inner wall, and when the desire is felt and the will is exercised, these muscles contract and the urine is expelled; but in paralysis of the bladder sensation is lost, and hence the sufferer is not conscious of the necessity for calling these muscles into play, and the muscles themselves, being under the influence of the nerves which have lost their power, cannot contract. It results, therefore, that urine comes into the bladder, distends it and over-fills it, and that when its distension is complete it dribbles away, just as water might dribble away from an over-filled cistern. So you must not think that when the patient is being continually wetted his bladder is necessarily acting; such a condition is a sign of over-distension, and calls urgently for relief by the surgeon's hands, and you should never fail to direct the medical man's attention to this condition of affairs when you meet with it.

When the haunch bones are broken the bladder, bowels, or other contents of the abdomen are peculiarly liable to be torn or bruised, and such injuries declare themselves by appropriate symptoms. If the bladder should be torn little or no urine escapes by the natural passage, as what enters it escapes by the rent, makes its way into the abdomen, and speedily sets up a highly destructive inflammation there; similar consequences follow from a tear of any of the bowels. Perhaps the most important fractures to you as nurses are the ones which involve the bones of the lower extremity, especially that part of the femur or thigh bone which lies in or near the hip joint. As we advance in life the neck of the thigh bone becomes more nearly set at a right angle than it is in younger life, and, as I told you before, the bones become more brittle with advanced age, and are more liable to break in consequence; so it happens that many old people sustain a fracture of the neck of the thigh bone from the smallest causes—tripping over a carpet, slipping over a piece of orange peel in the street—or slight injuries of this kind often result in an accident which makes cripples of them for the remainder of their days; for it is an anatomical fact that fractures of the thigh bone, which occur inside the capsule of the hip joint, do not repair with bone, and that such fractures result in mal-union, that is, in no bone union at all. In fractures about the hip joint the patient loses power over the whole limb, and it is noticed that the injured leg is shorter than its fellow; moreover, on lying down, the foot of the broken side falls outwards from its own gravity. The treatment adopted for these fractures is that of keeping the patient in bed for a few weeks, and then getting him up and allowing him to move about assisted by crutches and with some support to the injured leg; for it is found that if such people are kept in bed too long they lose muscular strength, and suffer much in health. The treatment of the other fractures of the thigh bone, at the hands of the surgeon, is by the application of a long splint on the outer side of the body and leg, with an opposing splint on the inner side of the leg, as I described to you just now, with the addition in most cases of an extension apparatus fixed to the feet to ensure the preservation of the proper length of the limb during the process of recovery. You so constantly see these extensions by weights, and splints applied to breakages of these bones, that I will not describe them.

A word or two about splints. You have to keep them padded and covered with some suitable soft material, so that they may be ready for the surgeon when he requires them. The best padding material is some form of cotton wool—gamgee-tissue is the best—but whatever your material may be, the great points are to provide soft padding extending well over the edges of the splint, and, where the splint is designed for covering parts which are likely to be soiled by any moisture, from the skin or elsewhere, to cover this layer of soft padding with some waterproof material. After the splints have been applied you must watch the patient from time to time to see that they do not press too tightly on the limb and so obstruct the circulation. The evidences of this —should it occur—will be complaints by the patients at first of pain and pressure, followed speedily by coldness and lividity of the parts which are exposed to view. If the tight bandages are not loosened now sensation will be lost in the limb below the point of pressure, and gangrene will result.

Now let us pass on to what takes place if the bones, either by neglect on the part of the surgeon, or by some defect in the constitution, do not set well after fractures. In the first place they may unite in any wrong position—side by side, at an angle, with cementing material connecting two bones—all of which conditions set up deformity in the limb where the bone has been broken; in the latter place we may have no union at all, or union by ligament, which will not get converted into bone, so that the patient is left with a false joint and a flail-like limb where all should be firm and strong as heretofore. The complications which arise in some fractures are mostly due to injuries sustained by nerves and blood vessels. When nerves are pressed upon or severed, tingling and partial loss of sensation and power, or complete paralysis of the parts below the injury, take place; when blood-vessels are divided, their contents escape into the flesh surrounding them, and the pulse below the injured part is lost; great bruising shows itself in the limb, and there is especial liability to the occurrence of gangrene or mortification.

### An African Health Resort.

Port Elizabeth is one of the pleasantest of South African towns—one in which a fortnight's holiday may be spent most enjoyably; for instance, by a tired nurse coming from some sandy desert "up country." It lies on the shore of Algoa Bay, which is a magnificent natural harbour, and the docks and pier are constantly alive and busy. All the mail steamers call, of course, and innumerable other vessels, and, as in Cape Town, representatives of almost every race under the sun, black or white, may here be met with.

The one disadvantage of the town is that, like Rome, it is built on hills; whether seven, I cannot say, but it seems more like seventy, and they are extremely steep. The ascents are very trying to the unfortunate horses, and not less so to the equally unfortunate foot passengers, for whom, in many places, short cuts are made by immense flights of steps cut in the solid rock. It is said, and no doubt with much truth, that all women who reside long in Port Elizabeth find their health seriously affected by the exertion of constantly ascending and descending these very steep hills. Those, however, who are merely spending a holiday there for the sake of the sea breezes can manage to keep mostly on level ground. There is a lovely walk along the sea wall or parade, made within the last few years, and it can be continued along the beach to the outlying district of Humewood, where there is a very fine new hotel; for some reason, however, this suburb, as it may be called, is terribly infested with flies during the summer, so much so as to make life hardly worth living there.

Port Elizabeth is built on bare rock. There is not an inch of soil anywhere, and the whole of the park, which is of a good size, is laid down with soil, every atom of which was carried from a distance in waggons. Here are planted grass, trees, and shrubs of all kinds, and in the centre are large conservatories, all this accomplished by sheer hard labour, in the face of two initial and very serious drawbacks, namely, want of soil and want of water. The town is supplied with water now from a river, which, like all South African rivers, is wont to fail in times of severe drought; but the engineering works by which the water is conveyed to the town are worthy of all the praise which has been bestowed on them.

In a word, Port Elizabeth is a thriving, pushing, enterprising place, which deserves to flourish and increase. The streets are broad, clean, and fresh, and in many cases planted with trees; the market place is a fine open square, on the upper side of which is the town hall, an imposing building containing the feather market, a large and very fine organ, and a public library.

There is a good-sized hospital in Port Elizabeth which stands on the summit of one of the aforementioned hills, a beautiful, breezy, open situation for a hospital, but rather hard of attainment. There is no asylum in the town, a fact which is to be regretted, inasmuch as the Grahamstown Asylum, already crowded, is the only one for all this part of the country.

For a convalescent requiring nothing but perfect rest and quiet, these may be found at New Brighton (so-called perhaps from the sharp contrast it presents to old Brighton), which is five miles from Port Elizabeth along the shore, and can also be reached by rail, as there is a siding ten minutes' walk from the hotel. This hotel constitutes New Brighton, for there are no other houses there, no shops, no anything, not so much as a fishing village. Even a boat cannot be procured. There is no gaiety except such as is supplied by an interminable expanse of sand and rocks, rainbow-coloured anemones, shells of every delicate shade, seaweed, bright scarlet and green. For the healthy and the gay, it is, therefore, a very dull spot; but it is an ideal refuge for the weary and worn, who only require rest, laziness, good food, and comfort. And on the days when returning health begins to infuse fresh vitality into the feeble frame, it is easy to spend a few hours, without much fatigue, amid the bustle and gaiety, the busy workers and the equally busy pleasure seekers, of the second seaport town in the Cape Colony.

### Christmas in Hospital.

### Royal Free Hospital.

Last week the students of the Royal Free Hospital gave an entertainment for the sisters, nurses, and patients of the hospital. The performance took place in the lecture-room, which was filled to overflowing, many members (both past and present) of the visiting and resident staff being also present. The first item on the programme was a tableau representing a Japanese tea-party and the Seasons. The grouping of these was most effective, especially of the former, in which dresses kindly lent by the D'Oyly Carte Company were worn. The interval between the tableaux was filled up by a topical dialogue, in which many little peculiarities of the staff were taken off. It was heartily enjoyed by those who understood the allusions, and perhaps not least by those at whom they were directed. Last followed the performance of the farce, "Ici on parle Français," which was acted with great spirit and elicited much applause. The intervals between the pieces were filled up with music, and the proceedings ended with the singing of "God Save the Queen."

### The National Hospital for Diseases of the Heart, Soho Square.

At the above hospital an entertainment was given on Christmas Eve by Mr. and Mrs. Stalman and friends in the large female ward, where a temporary stage was erected. Two farces, "A Case for Eviction" and "A Naval Engagement," were capitally acted and afforded great amusement to the patients. During the interval some pianoforte pieces were performed by the Rev. Mr. Walton, curate of St. Anne's, Soho. At the conclusion of the second farce it was announced that an old gentleman known as Father Christmas had paid a flying visit and had left a large basketful of presents. These, which consisted of useful articles of clothing for the men and women and toys for the children, were distributed. After this function a selection of carols were sung by the boys of St. Ann's choir in their well-known good style. A cordial vote of thanks to the entertainers closed a very enjoyable evening. The decoration of the wards was undertaken by the nurses and was most successful.

### Lincoln County Hospital.

All the patients who were well enough at this hospital were regaled with pheasants, rabbits, and plum pudding, after which the men were allowed to smoke in the out-patient room. In the women's ward a tea party was held, followed by an exhibition of Mrs. Jarley's waxworks, kindly arranged by the nursing staff. This week a similar entertainment was held in the male ward. The children enjoyed a tea party, followed by a Christmas tree, after which they joined in various games.

### The Royal Infirmary, Bradford.

On Christmas Day the Mayor of Bradford provided dinner at various charitable institutions and presented them with a gift of £100. The Royal Infirmary reaped the lion's share. The nurses did their part by prettily decorating the wards. Dinner was served at noon, and a large number of ladies and gentlemen kindly helped with the carving and waiting. The presence of these well-wishers conduced much to the pleasure of the 183 inmates who partook of it. This is the second occasion on which the Mayor, Mr. Thomas Speight, has provided the Christmas feast. Gifts were distributed later. The best feature in this enjoyable entertainment is the large number of people who have spent time and thought in co-operating with the hospital authorities to make Christmastide happy.

### Our Christmas Gifts.

THE fog was so dense on Thursday that the distribution of clothing to the hospitals from our Needlework Distribution Fund was put off until Friday. This was a cold, bleak day, and the fog fell heavily in the afternoon, but lifted later. It enabled one to realise from personal experience how utterly miserable the want of light and warmth can make even the well fed and warmly clad. All through the long, dreary drive that took us north and east there were abundant evidences of poverty on every side, and every time we entered a hospital we were greeted with a gratitude that made us wish we had a cab-load of parcels for each instead of only one to divide amongst so many. Our readers will notice by the list given last week that the poorer and less-known hospitals were chosen, and they would have been sure that the choice was a wise one could they have obtained a glimpse of the various institutions to which their gifts went. In some the wards and the hospital were filled with the confusion of decorating in a thick fog by gaslight, but neither the workers nor the patients seemed aware how uncomfortable it looked to an outsider. It was impossible to see faces, but the air was filled with laughter and happy chatter. In two a red robe trimmed with cotton wool, lying in the matron's room, betrayed preparations for Father Christmas's visit, and we could not help feeling glad that our contributions to his budget of gifts were ready in time.

As we passed on our way a resolution sprang up, and with each succeeding visit it grew stronger. It was, we must do much better than this next year. If so much can be done by comparatively few nurses, how much can we not do if we all put our backs into it? At present, the nurses in hospital bear the brunt of the greater part of the making of Christmas joy for their poor patients, and it is only fair that others who have so much time and so many golden opportunities should use them to strengthen their sisters' hands in this. All and every sort of thing is wanted, and it seems to us that if our readers would only co-operate loyally and all the year round we might do very valuable work in this way. For instance, some nurses make frocks, some pinafores, some shoes, or shirts, or petticoats. Now, if one would send a vest, another a shirt, another a petticoat, another a frock, another a pinafore, another a pair of shoes, say once, twice, or thrice, or even four times a year, just so many persons would go out of hospital with a decent, comfortable suit of clothing. There is no necessity for the material to be absolutely new. Listen to the history of a dressing-gown. It was home made, of soft thick blue flannel. It served as a dressing-gown for quite eighteen months, and then it became a bed wrap. It was still soft and warm, but the blue had faded to a most æsthetic shade. One day the owner visited an old servant and found her in tears. One baby was ill, her husband out of work, and another baby was expected. "It is as much as I can do to scrape along now, and I cannot think where I am to get its clothes." But fortunately there was the old dressing-gown. It was cut up and made very pretty little garments, "almost better than new, because it was soft and would not shrink any more," said the mother, with the greatest satisfaction. As it happened, the baby only lived a few days, but the garments prepared for it out of the dressing-gown were converted by the mother's skilful needle into winter vests and petticoats for her other children. A few weeks afterwards the father got good work, and all was well again, so the old dressing-gown had done its work well from beginning to end. Nurses, have you not got nice old flannel garments lying by? A pattern, a few hours' work, and they will be rescued from the realms of waste material to carry comfort where it is greatly needed.

Will each nurse who wishes to join in this work send us a postcard bearing her name and address and *nom de plume*, and will she also say what garment she will send, how many of them, and when we may expect it or them? We shall then make arrangements to receive and distribute the things as regularly as we can, and will acknowledge each contribution in the "Mirror." It rests with the nurses to make this a success if they will.

### Hypnotism Across the Herring Pond.

HYPNOTISM has found little favour so far in England. Some medical men are devoting all their time to its study, but so much difficulty lies in acquiring the art, and so many dangers in practising it, that its use is by no means common. It has, possibly a great future, but before it can be commonly adopted as a therapeutic agent there is much to be learnt, both respecting its nature and its subsequent effects.

One thing recognised with regard to hypnotism by all medical men is the danger of its use by ignorant or unscrupulous persons, and also that the only people who can employ it safely and with benefit are honourable medical men. The art of hypnotism, therefore, stands in the same relation to the world of sickness as a comparatively unknown and probably dangerous drug.

American nurses are progressive, and, from an article in the *Trained Nurse* for November, are evidently preparing to rush gaily in where the more learned profession fears to tread. Our readers will note in the extract that it is a lady nurse and a male patient, and they can more easily imagine Mrs. Grundy's censures on the proceedings than we can describe them. After the patient is comfortably in bed "seat yourself beside him and request him to fasten his eyes upon yourself. Tell him that his attention is not to wander from you for a moment, that he will always, even though he should go sound asleep, hear you speak to him, but that he will not rouse himself sufficiently to throw off this sleep condition, and will remain passive throughout." After securing the patient's undivided attention for three minutes, "suggest to him that he is beginning to feel a drowsiness steal over him and his eyes are growing tired, and that in a little while he will go quietly to sleep." Then follow directions for closing the eyes and making various passes and suggestions.

It is erroneously stated that this sleep differs in no way from natural sleep. The hypnotic "sleep," however, is one which leaves the subject very much under the control of the operator. Whereas in natural sleep, in spite of all the vagaries and dreams of the brain, the will is still master of its own temple, in that induced by hypnosis another will rules in the throne of the abdicating monarch, a condition of things that prepares the scene sometimes for tragedy, sometimes for scandal.

### Appointments.

### MATRONS.

THE CHILDREN'S HOSPITAL, MIDDLESBOROUGH.—Miss Laura Denniss was appointed Matron of this hospital on December 16th. She was trained at the General Hospital, Nottingham, where she remained nearly four years. On leaving Nottingham she was appointed sister of the female medical and children's wards of the Blackburn and East Lancashire Infirmary.

ROYAL HOSPITAL FOR DISEASES OF THE CHEST, CITY ROAD, E.C.—Miss Agnes Megginson was appointed Matron of this hospital on December 20th. She was trained at St. Bartholomew's, and has been for seven years sister of the "Faith" ward of that hospital.

### Wants and Workers.

SEMPER PARATUS asks to be informed of any suitable place, sanatorium or hospital, where a nurse with incipient phthisis might be received. She could afford to pay £1 weekly.

### A Book and its Story.

"MEMORIES OF THE CRIMEA."

The writer of this little book* was one of a band of Roman Catholic Sisters of Mercy who went out as nurses to the Crimea. On her return to England she was attached tc the Hospital of S. John and S. Elizabeth, in Great Ormond Street, and it may be remembered that since last winter she was, in recognition of her services in the East, decorated by the Queen with the order of the Royal Red Cross. A melancholy interest attaches to these memories from the fact that only last month the author died, in her 82nd year, being then one of the few survivors of the ladies who emulated Miss Florence Nightingale's zeal in her mission of healing and mercy to the sick and wounded. This volume, though it contains but little which is new, gives a modest and unpretentious account of some very efficient work done in the hospitals by "Sister Mary" and her companions. The military arrangements of the British force in the winter of 1854-55 were most seriously hampered by the want of a proper organisation for dealing with the treatment of the sick and wounded. The medical department of the army had, during the long peace which followed the Battle of Waterloo, become disorganised; and the hasty arrangements which the Medical Staff attempted to make at the outbreak of the war with Russia proved totally inadequate. Immense stores of medicine, instruments, and hospital fittings had been provided; but in every place to which the sick and wounded were conveyed there was confusion, overcrowding, and a complete want of system. At this moment, when one B.itish army is advancing over the sands of Egypt to the desolate regions of equatorial Africa, and another is facing the difficulties of mountain warfare amongst the stupendous precipices of the North-West Frontier of India, we may well be thankful to know that the lessons of the Crimean War have not been forgotten, and that valuable lives are no longer endangered by the want of medical attendance in the field. During the terrible period of the Crimean War, when every morning the papers contained a long list of killed and wounded, side by side with the news which brought mourning into so many homes were to be found columns filled with indignant remonstrances against the unnecessary sufferings to which our sick and wounded soldiers were exposed. It was when the shortcomings of the then existing medical system were being denounced as a national disgrace, that, in a fortunate moment, the Government recognised the fact that women were the best nurses. Miss Nightingale became superintendent of hospital nurses at Scutari, and started for the East with her company of fellow-workers. Others followed, and amongst these were Sister Mary Aloysius, and the Roman Catholic sisters.

"One morning," she says, "as the sisters were assembled for morning lecture in the Communion Room of the Convent of Mercy, Carlow, the Rev. Mother read a letter received by the morning post from the parent house of the sisterhood. The letter was an invitation to some of the nuns to go as nurses to the seat of war, and it was nobly accepted. "The appeal from the East," says Sister Mary, "no Sister of Mercy could resist, and highly privileged did those deem themselves who were chosen for the enterprise. The hospitals were represented as filled with the dead and dying. The trenches were filled with the stiffening corpses of many a frozen warrior. . . . Neither linen nor lint could be found, orderlies were their only nurses. Our allies did not suffer in this way, they summoned their sisters on the first appearance of sickness, and the questions were constantly asked, 'Are there no nurses in England? Can the women do nothing for us in this fearful emergency?'." The emergency was, indeed, fearful; for in the military hospital at Scutari alone there were 2,500 sick soldiers and only 10 physicians.

On December 2nd, 1854, they started, these good nuns, and at London Bridge formed part of as curious a group as London Bridge had witnessed. The ladies and paid nurses wore the same costume, and a very ugly one it was. It seemed to be contract work, and all the same size, so that the ladies who were tall had short dresses, and the ladies who were small had long dresses. They consisted of grey tweed wraps, worsted jackets, white caps, and short woollen cloaks, and, to conclude, a frightful scarf of brown holland embroidered in red, with the words "Scutari Hospital." That ladies could be found to walk into such a costume was certainly a triumph of grace over Nature. This party was under the charge of Miss Stanley, sister of Dean Stanley, who afterwards joined the Church of Rome. Adrrive at Scutari, they were told "The War Office had made a mistake in sending the party—no room for them!" In the long run, however, five of the sisters, Sister Mary Aloysius being one of the number, were sent to Scutari. "Where shall I begin, or how shall I ever describe my first day in the hospital at Scutari? Vessels were arriving, and the orderlies carrying the poor fellows, who with their wounds and frost-bites had been tossing about on the Black Sea for two or three days, and sometimes more. Where were they to go? Not an available bed. They were laid on the floor, one after another, till the beds were emptied of those dying of cholera and every other disease."

Details of the cases of cholera and the treatment, which seldom was of any avail, are very dreadful; and the horrors did not end with the death of the patients. "It was said that the graves were not made deep enough, and that the very air was putrid. There were no coffins—canvas and blankets had to suffice." Then there were cases of frost-bite, when wounded soldiers were carried in with their flesh and clothes frozen together. "Far, far worse and more painful were these than the gun or sword wounds; and what must it have been where they had both?"

The Sisters remained at Scutari till October, 1855, when they were sent to Balaclava. There, close to the front, they were in the midst of all the horrors of war. Men were dying on all sides from wounds and disease, cholera and typhoid fever being the most fatal; and a report from the deputy-purveyor to the War Office, dated December 24th, 1855, bears testimony to the work of the women nurses. "The medical officer," he says, "can safely consign his most critical cases to their hands. Stimulants or opiates ordered every five minutes will be faithfully administered, though the five minutes' labour were repeated uninterruptedly for a week." Two of these devoted women fell victims to their duty. "Sister Winifred" died on their third day at Balaclava, of cholera; and at the end of February, 1856, "Sister Mary Elizabeth" followed her to the grave. In the following spring, as soon as peace was concluded, the sisters left the Crimea. Their departure appears to have been hurried; and the story reads as if there had been some misunderstanding. There had been complaints that the sisters had interfered in matters of religion with patients who were not Roman Catholics. The account given by Sister Mary of the facts is somewhat vague. But what the difficulty was is now of no importance; and there can be no manner of doubt that the Irish nurses fully deserved the cordial thanks which they received from all who witnessed their labours of love. This record of their services, though, as we have said, it adds but little that is new to the oft-told story of the Crimean War, will be read with interest, especially, as Dr. Fahey remarks in the preface to the volume, "at a time when the ominous echoes of war resound once more through Europe from Eastern battle-fields."

* "Memories of the Crimea." By Sister Mary Aloysius. (Burns and Oates, London. 1897.)

### Everybody's Opinion.

[Correspondence on all subjects is invited, but we cannot in any way be responsible for the opinions expressed by our correspondents. No communication can be entertained if the name and address of the correspondent is not given, as a guarantee of good faith but not necessarily for publication, or unless one side of the paper only is written on.]

### A CANCER CASE.

It is with much pleasure that we insert the replies of so many correspondents to a "District Nurse" on this case. They are most instructive, and beside they illustrate how beneficial a nurse's experience is to nurses. No one can help a nurse in many ways quite so much as a fellow-nurse, and we trust that our readers will remember this and come forward and co-operate with each other by means of the "Mirror," and not leave the editors to try and solve all problems alone. Will the correspondents on the cancer case kindly accept the editor's thanks, as well as those of a "District Nurse's," for their prompt and courteous response to last week's query.—Ed. *T.H.*

"A Matron" writes: In answer to "District Nurse," I would suggest small pillows filled with sawdust or bran. These can be frequently renewed, the soiled sawdust being burned and the covers washed. Any carpenter will supply sawdust at a cheap rate. There is a special urinal sold for such cases, with a long tube attached, which falls into a utensil at the side of the bed. They are rather expensive, but might be obtained through the Surgical Aid Society. I have an old one I would send "District Nurse" to see, if she likes to write to me and pay postage.

"A Fellow District Nurse" writes: Would a district nurse not find pads of teased oakum or unbleached cotton wool in butter muslin inexpensive and a comfort to her patient? I find oakum very good in pads, and often use it in district work—of course, with just a layer of wool over it to make it soft. Could she not also keep a little vessel in position under the patient so as to receive some of the urine? A toothbrush dish I have sometimes found useful in such cases. If these hints are of any use to a fellow worker I shall be very glad.

M. Dee writes: In answer to your question of December 18th, I have had many similar cases. Those with bedsores were usually not made known to me until too late, for I generally find, with the free use of soap and water, also methylated spirits and dry pads, you can usually keep the patients free from bedsores. I always wash and change the draw-sheet daily; the pads, I leave word with the patient's friends to change and burn at once when wet, *i.e.*, if I only visit the case once daily. My pads I make in the following manner: First, I prepare two layers of cotton wool, then wood wool (or carbolised tow if the smell is offensive), and in the middle I put scraps of old calico, linen, flannel or flannelette—anything that is of no use and will absorb. Afterwards cover the whole with a thin muslin, which I get in large quantities for a copper or two from the foreign meat shops or large grocers where they sell rolled bacon. When this is washed and boiled it makes a splendid covering. The pads I make about 12 inches square. The cotton and wood wools cost about one shilling a pound, and half a pound of each goes a long way. If "District Nurse" will send me her address I will send her one to look at, if I have not explained my meaning clearly.

### THE ROYAL BRITISH NURSES' ASSOCIATION.

Dr. W. Bezly Thorne writes: In your account of the special general meeting of the Royal British Nurses' Association, which was held on the 11th inst., the President of the Incorporated Medical Practitioners' Association is reported to have said that the body over which he presides had "helped the Nurses' Association at first, and, as a sort of compensation for that help, the Nurses' Association had agreed to make the president an *ex-officio* member of the (executive) committee." Dr. Hugh Woods went further, and stated that the alleged agreement was the result of some kind of barter for value to be received in the form of support, and added, in answer to my inquiry, that the transaction was conducted at a meeting of his association. That allegation would be sufficiently important if it had been made in the heat of discussion only; but Dr. Hugh Woods had already advanced it at a meeting of the Royal British Nurses' Association shortly after he entered on the office which he now holds, and he repeated it in a circular letter which he, in his official capacity, addressed on October 15th last to the nurse members of the nursing corporation, in the following words: "The officials propose that the association should break its promises . . . and remove the presidents of those bodies from the *ex-officio* seats which they now hold." Now the meeting referred to by Dr. Hugh Woods was attended by Dr. Bedford Fenwick, myself, and another member of the British Nurses' Association; and, as one of the three, I wish to inform those whom it may in any way concern that the allegation of Dr. Hugh Woods is not a true one. In confirmation of my assertion, I would call attention to the following points: Neither of the governing bodies of our association was informed of our intention to attend the meeting of the Practitioners' Association; we attended as private members at the solicitation of Dr. Bedford Fenwick; we neither possessed, nor pretended to have been invested with, authority to represent or to act for our association; we neither negotiated, bartered, agreed, nor promised—had we done so, we should have acted *ultra vires* and constituted ourselves impostors. What we did do was to explain the objects of our association and its constitution, pointing out more especially that registered medical practitioners of all ranks and classes were represented on its governing bodies, and that the medical profession was thus enabled to exercise a powerful and necessary influence on the administration of what was about to become the only legally organised body of nurses. Those statements satisfied the members of the General Practitioners' Association that it would be in accordance with their views and interests to support rather than oppose the petition of the Nurses' Association for a Royal charter, and they voted accordingly. Such being the case, I challenge the president of the Incorporated Medical Practitioners' Association to substantiate or to withdraw his allegation that promises were made and that an agreement was entered into.

### The Queen's Message to Miss Lumsden.

We think it will be interesting to record some of the work done by Miss Lumsden, which has led Her Majesty to express her appreciation of her services. The Royal Aberdeen Hospital for Sick Children was started in 1887, when Miss Rachel Frances Lumsden, who had been trained and had worked in the Hospital for Hip Disease, Queen Square, London, the Great Ormond Street Children's Hospital, and the King's College Hospital, was asked to undertake its organisation and superintendence. In 1886 the directors of the Aberdeen Royal Infirmary invited her to reform and reorganise the working of that institution, which had fallen far behind the requirements of the age. This she agreed to do, her post at the Sick Children's Hospital being taken by Miss Katharine Lumsden, the present hon. superintendent. In 1895, when the new surgical pavilion of the Royal Infirmary was opened by Her Royal Highness the Princess Louise, Marchioness of Lorne, the wards in that block were named, one of them being called the Rachel Frances Lumsden Ward. In the present year, on Miss Lumsden's retirement from her twenty years' gratuitous hospital work in Aberdeen, the following gracious message has been received from her Majesty: "Her Majesty has learned with interest and deep appreciation the great and valued services which, during upwards of twenty years, Miss Lumsden has given with untiring zeal and self-denial to the sick and suffering of the poorer classes in Aberdeen."

### Presentations.

A very handsome entreé dish was presented on Christmas Day by the staff of the Blackheath and Richmond Nursing Institution to Mrs. Smith and Miss Duncan, with a much-valued expression of kindly feeling.

### For Reading to the Sick.

THE NEW YEAR.

"FORWARD out of darkness, forward into light."

**Verses.**

Onward and upward still our way,
With the joy of progress from day to day;
Nearer and nearer every year
To the visions and hopes most true and dear!
Children still of a Father's love,
Children still of a home above!
Thus we look back
Without a sigh on the lengthening track.

Learn the mystery of progression duly,
Do not call each glorious change Decay;
But know we only hold our treasures truly
When it seems as if they pass'd away!
Nor dare to blame God's gifts for incompleteness!
In that want their beauty lies; they roll
Towards some infinite depth of love and sweetness
Bearing onward man's reluctant soul.

—*A. Proctor.*

Take my life, and let it be
Consecrated, Lord, to Thee;
Take my moments and my days,
Let them flow in ceaseless praise;
Take my hands and let them move
At the impulse of Thy love;
Take my feet, and let them be
Swift and beautiful for Thee;
Take my voice, and let me sing
Always, only for my King;
Take my lips, and let them be
Filled with messages from Thee.

Take my silver and my gold,
Not a mite would I withhold;
Take my intellect and use
Every power as Thou shalt choose;
Take my will and make it Thine,
It shall be no longer mine;
Take my heart, it is Thine own,
It shall be Thy royal throne;
Take my love, my Lord, I pour
At thy feet its treasure-store;
Take myself and I will be
Ever, only all for Thee!

—*F. R. Havergal.*

The man who consecrates his hours
By vig'rous effort, and an honest aim,
At once he draws the sting of life and death.

—*Young.*

From henceforth thou shalt learn that there is love
To long for, pureness to desire, a mount
Of consecration it were good to scale. —*J. Ingelow.*

**Reading.**

Here we offer a present unto Thee, O Lord, ourselves, our souls and bodies, to be a reasonable, holy, and lively sacrifice unto Thee. And although we be unworthy through our manifold sins to offer unto Thee any sacrifice, yet we beseech thee to accept this our bounden duty and service.

I beseech you, therefore, brethren, by the mercies of God, that ye present your bodies a living sacrifice, holy, acceptable unto God, which is your reasonable service."—*Romans* xii. 1.

God helps us in our prayers, but He does so in proportion as we admit his aid in the rest of our life. We cannot pray as we ought unless we live as we ought. Our prayers will partake of our other infirmities. We cannot at once collect ourselves and become other men in the Presence of God from what we are just before.—*E. B. Pusey.*

Meanwhile, do the work which concerns you at the present moment; go on quietly with your spiritual exercises, give yourself up many times each day, both heart and mind, into God's hands, commending your work humbly to Him.—*S. Francis de Sales.*

### Notes and Queries.

**The contents of the Editor's Letter-box have now reached such unwieldy proportions that it has become necessary to establish a hard and fast rule regarding Answers to Correspondents. In future, all questions requiring replies will continue to be answered in this column without any fee. If an answer is required by letter, a fee of half-a-crown must be enclosed with the note containing the enquiry. We are always pleased to help our numerous correspondents to the fullest extent, and we can trust them to sympathise in the overwhelming amount of writing which makes the new rules a necessity. Every communication must be accompanied by the writer's name and address, otherwise it will receive no attention.**

*Certificate as Mental Nurse.*

(103) Will you kindly tell me the best way of obtaining training as a mental nurse with view of getting certificate for a lady who has had some experience? Also if an arrangement could be made for payment afterwards?—*J. C.*

Write to the Medical Superintendent, Northampton County Asylum, Berrywood, near Northampton.

*A Probationer's Books.*

(104) As I wish to get some books for a friend, who is a probationer in a surgical and nursing home, I should feel greatly obliged if you would kindly let me know, through the medium of your paper, what books you would recommend on general and surgical nursing as being most suitable and useful to her?—*Books.*

"The Nurses' Dictionary of Medical Terms and Nursing Treatment" (Honnor Morten), 2s., post free. (2) "Surgical Ward Work and Nursing" (Miles), 3s. 6d. (3) "THE HOSPITAL Nurses' Case Book," price 6d. (4) "The Matron's Course," price 1s. (Miss S. E. Orme). (5) "Notes on Surgery for Nurses" (Joseph Bell), 2s. 6d. From the Scientific Press, 28 & 29, Southampton Street, Strand.

*Training.*

(105) 1. Can you tell me some hospitals in London that take probationers at 21 years of age? 2. Which of these said hospitals is considered best for training? 3. Do they take paying or non-paying probationers? Is it better to pay a premium? What are the advantages, generally speaking, for a paying probationer in regard to a non-paying probationer?—*Florence Wright.*

1. Only in the children's and special hospitals. 2. A year's training in a children's hospital is only useful as being a good preparation for work in a general hospital later. 3. Many hospitals take paying probationers. The advantages vary with the different institutions. For instance, at Guy's a paying pupil who gives satisfaction is allowed to become a probationer at the end of a year. From this you will see that there is very little gain, as a rule, in paying. The advantages often lie only in extra creature comforts. Miss Honnor Morten's "How to Become a Nurse" (Scientific Press, 28 & 29, Southampton Street, Strand, London, W.C., 2s. 6d., post free) would give you some useful hints.

*Princess Christian's Trained Nurses.*

(106) Will you kindly tell me how, or through what influences, I can belong to Princess Christian's Private Nursing Home? I am a member of her Army Nursing Reserve Corps.—*Nelly.*

Application may be made to the Lady Superintendent, H.R.H. Princess Christian's Trained Nurses, 1, Clarence Villas, Windsor.

*Membership of R.B.N.A.*

(107) Will you kindly inform me if only fully-trained—that is, nurses who have had three years' hospital training—nurses are eligible for membership in the Royal British Nurses' Association? I am a private nurse, a certificated masseuse, and hold the L.O.S. If I can become a member what are the terms—subscription, entrance fee, &c.?—*Nurse T.*

Only nurses with a three years' certificate can become members of the R.B.N.A.

*Fever Nursing in Cape Town.*

(108) I would like to know if there is an opening in Cape Town for fever nurses. I have had four years' experience in good training fever hospitals, and am 25 years of age? From where could I get particulars of same?—*Nurse C.*

There is almost certain to be an opening, but you must go out to definite work, and it is difficult to hear of at such a distance. Perhaps if you wrote to the Matron, New Somerset Hospital, Cape Town, South Africa, she could tell you.

*A Book on Massage.*

(109) Will you kindly recommend to me a good book on massage?—*F. R.*

"The Art of Massage," by A. Creighton Halle, is highly recommended, price 6s., from the Scientific Press, 28 & 29, Southampton Street, Strand, London, W.C.

*A Private Nursing Home.*

(110) I should like to know if I could get a notice of my home in THE HOSPITAL?—*E. H. B.*

We only give a notice of homes after personal inspection. If one of our representatives should be in the neighbourhood of your home, we shall be pleased to pay it a visit.

**ANSWERS INVITED.**

*Bed Sores.*

(111) What is the best thing to prevent bed sores? I was taught at Brownlow Hill Infirmary before the skin was broken, to wash the back well with soap and water, to rub it with methylated spirit, and then to dust it with zinc powder. Now I am told that alum water and a little starch powder is just the same thing, and am directed to pound some starch in a mortar for this purpose. I cannot get the powder as fine as we buy. I have two patients with terribly broken sores. Charcoal poultices have been ordered for them, and I am supplied with it in lumps. Can I prepare it satisfactorily myself? Also, should a nurse be asked to do such black work?—*M.*

